# Implications of Gut Microbiota in Neurodegenerative Diseases

**DOI:** 10.3389/fimmu.2022.785644

**Published:** 2022-02-14

**Authors:** Haoming Zhang, Yijia Chen, Zifan Wang, Gaijie Xie, Mingming Liu, Boyu Yuan, Hongxia Chai, Wei Wang, Ping Cheng

**Affiliations:** ^1^ Innovative Institute of Animal Healthy Breeding, College of Animal Sciences and Technology, Zhongkai University of Agriculture and Engineering, Guangzhou, China; ^2^ School of Life Science, Fudan University, Shanghai, China; ^3^ Key Laboratory of Zoonosis Research, Ministry of Education, Jilin University, Changchun, China; ^4^ State Key Laboratory of Oncology in South China, Collaborative Innovation Center for Cancer Medicine, Sun Yat-sen University Cancer Center, Guangzhou, China

**Keywords:** gut microbiota, neurodegenerative diseases, gut–brain axis, blood–brain barrier, microbial molecules

## Abstract

The morbidity associated with neurodegenerative diseases (NDs) is increasing, posing a threat to the mental and physical quality of life of humans. The crucial effect of microbiota on brain physiological processes is mediated through a bidirectional interaction, termed as the gut–brain axis (GBA), which is being investigated in studies. Many clinical and laboratory trials have indicated the importance of microbiota in the development of NDs *via* various microbial molecules that transmit from the gut to the brain across the GBA or nervous system. In this review, we summarize the implications of gut microbiota in ND, which will be beneficial for understanding the etiology and progression of NDs that may in turn help in developing ND interventions and clinical treatments for these diseases.

## 1 Introduction

Neurodegenerative diseases (NDs) including Alzheimer’s disease (AD), Parkinson’s disease (PD), Huntingdon disease (HD), and multiple sclerosis (MS) are characterized by the progressive loss of neurons that is associated with neurotoxic etiological substances in the brain and the surrounding organs. The maximum human life span has expanded because of the improvement in nutrition and health care with the development of the economy and technology. However, the incidence of NDs increases with age, generating increasingly severe burdens to society (1, 2). Unfortunately, because of the unclear pathogenesis of these diseases and the complexity of the nervous system, an effective treatment is lacking, although several clinical trials are ongoing.

Diverse microbes including bacteria, archaea, viruses, and various eukaryotes such as fungi and protozoa are present in different ecological niches in the gut and are collectively known as the gut microbiota (3). The gut microbiota profoundly affects several aspects of host physiology, including nutritional metabolism, anti-infection, immune system, and nerve development (4, 5). Rapid industrialization, urbanization, and development in food and medical technology, such as increasing intake of fast food, cause the gut microbiota to confront a different habitat, and thus, it has become more vulnerable than before (6). Recently, the importance of gut microbiota has emerged because of its vital role in NDs and in modulating the differentiation, maturation, proliferation, and activation of tissue-resident immune cells in the central nervous system (CNS) (7–11).

Gut–brain axis (GBA) participates in the bidirectional communication between the gut and the brain *via* neurotransmitters and various metabolites (12, 13). In this review, we summarize the possible pathophysiological roles of the microbiota in NDs. Furthermore, we focus on the potential of microbiota composition and metabolites as novel therapeutic interventions for these chronic diseases.

### 1.1 Gut Microbiota

Gut microbiota that consists of various dynamic microorganisms establishes a symbiotic relationship with the host. The metabolic activities and interactions of gut microbiota affect normal physiology and susceptibility of the host to diseases (5, 14). The differences in pH, immune factors, and digestive enzymes in the gut are responsible for the diversity and individual differences at the bacterial strain level (15). Moreover, each individual harbors distinct microbial community that results in the formation of a stable and resilient state (14). In an adult gut mucosa, *Bacteroidetes* and *Firmicutes* are the predominant phyla, whereas the abundance of *Actinobacteria*, *Proteobacteria*, and *Verrucomicrobia* is low (16, 17).

Gut microbiota can directly affect human health by secreting microbial components such as vitamins, essential amino acids, and lipids (9, 13, 18). These components may be involved in the GBA *via* neural, endocrine, and immune signaling pathways and thus affect physiological functions such as gastrointestinal barrier, nutritional metabolism, immune response, and neurological development associated with aging (19–22).

### 1.2 Correlation Between Gut Microbiota and Neurodegenerative Disease

The gut microbiota is considered essential for brain physiological processes such as myelination, neurogenesis, and microglial activation; regulation of human behavior; and affecting mental processes such as mood and cognition (9, 23, 24). Moreover, the gut microbiota is highly sensitive to external lifestyles such as diet, sleep deprivation, circadian rhythm disturbances, chronic noise, and sedentary behavior, which are also considered the risk factors for some NDs (6, 25–31). The gut microbiota is critical for maintaining a healthy functional state of microglia, which is necessary to prevent neurodevelopmental abnormalities and NDs (8, 32–34). Clinical trials have confirmed the crucial role of microglia activation in AD pathology (22, 35, 36).

### 1.3 Gut Microbiota–Brain Communications

#### 1.3.1 Gut–Brain Axis

**Figure 1:**
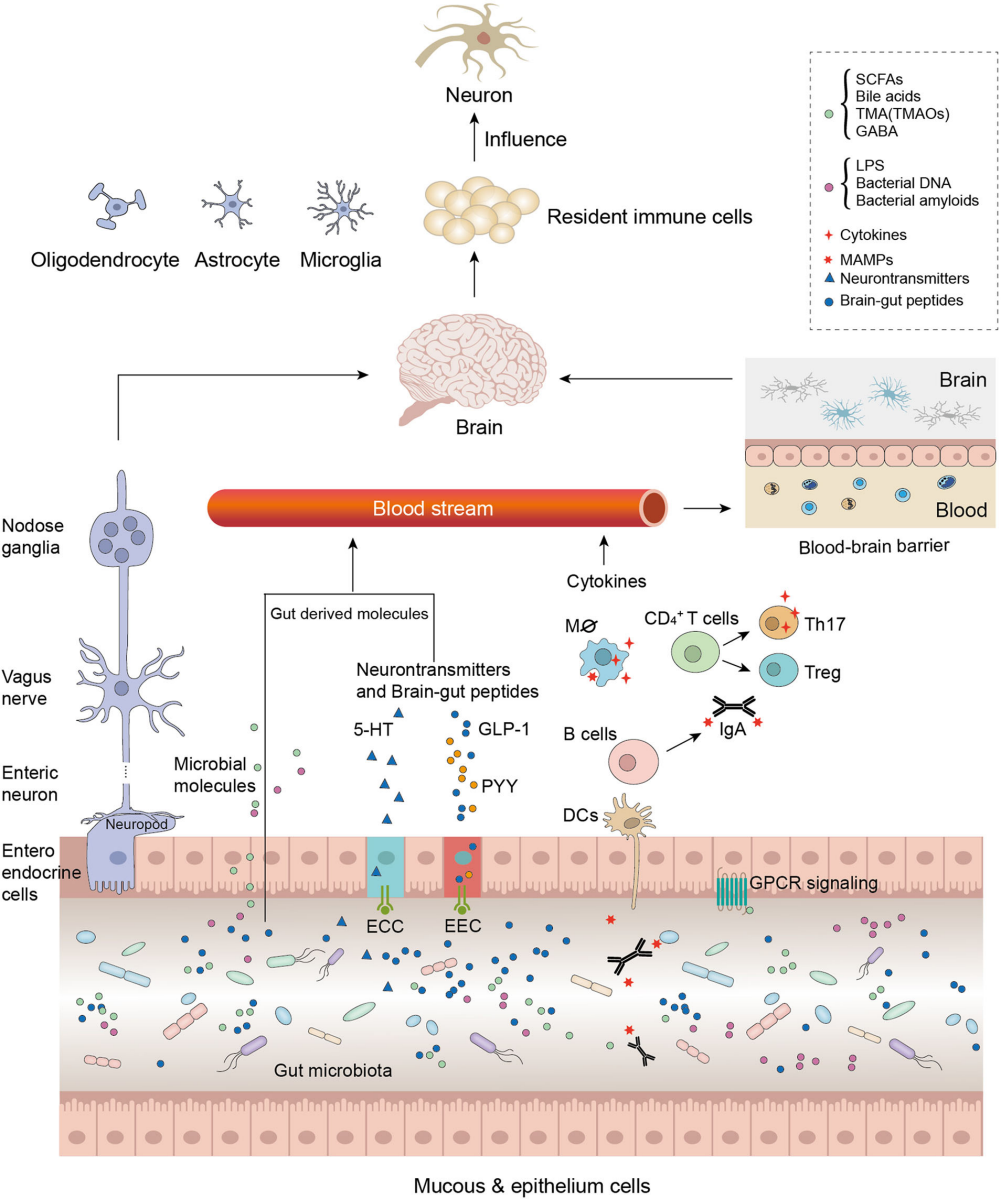
Microbiota modulates the gut–brain axis through its secretions, including microorganism-associated molecular patterns (MAMPs) and microbial metabolites. As the intestinal permeability decreased due to the microbial molecules, these molecules were involved in generalized gut–brain signaling, such as the immune-modulating pathway, endocrine signaling pathway, the neural signaling pathway, and the neuroendocrine signaling pathway. Neurotransmitter-like substances such as gamma-aminobutyric acid (GABA) directly influence the central nervous system (CNS) through nerve pathways; other gut-derived substances such as MAMPs and short-chain fatty acids (SCFAs) influence the CNS by decreasing blood–brain barrier (BBB) permeability. Furthermore, these microbial molecules activate immune resident cells or neuron cells, which accelerate neurodegenerative disease (ND) physiopathology.

GBA refers to the communication between gut microbiota and the brain and involves multiple physiological processes, which are strategic points in maintaining the homeostasis of the gastrointestinal (GI) tract, CNS, and microbial systems (**Figure 1**).

The pathways comprise the vagus nerve and the neural, endocrine, and immune systems that exert direct or indirect effects by stimulating the release of chemical transmitters such as microbial hormones and metabolites (37, 38). Therefore, these systems involved in GBA regulate many functions, such as regulation of brain activity and emotions, immunomodulation, energy balance, and activation (39–41). The microbiota as a link between mental health, cognitive brain centers, and peripheral intestinal function has garnered considerable scientific attention. Studies have reported that the disruption of GBA may be associated with mood disorders and dysbiosis of gut microbiota (40, 42), and the microbiota may affect the anxiety and depressive behavior *via* the GBA (43). Depending on the disease severity, specific microbiota alterations are found in autistic patients (44).

#### 1.3.2 Blood–Brain Barrier and Gut-Derived Molecules

The blood–brain barrier (BBB) is an anatomical, functional structure that separates blood from brain tissues and cerebrospinal fluid; it is composed of pia mater, choroid plexus, cerebrovascular, and astrocytes (45–47). The BBB serves as a gateway for the passage of many crucial substances required for CNS functioning and secretes substances into the blood and brain that are crucial for maintaining the CNS homeostasis. Moreover, the BBB could also limit the transport of gut-derived molecules into the brain (48). Vulnerable BBB is caused by aging and may induce cerebrovascular inflammation and CNS disorder (49–52).

For example, microorganism-associated molecular patterns (MAMPs) play critical roles in the structural integrity and essential cellular functions in microorganisms (53). When MAMPs are accidentally enhanced or decreased, acute or chronic inflammation associated with various neurological disorders is induced (54).

Several microbial molecules such as lipopolysaccharides (LPS), short-chain fatty acids (SCFAs), trimethylamines (TMAs), and vitamins are associated with the permeability of BBB (55–57). These molecules could act on BBB to directly affect brain neurons or stimulate the immune and endocrine systems to protect against neuroinflammation or neurodegeneration.

LPS is a crucial component of the outer membrane of Gram-negative bacteria. It is one of the most extensively studied components of bacterial immune stimulation, which can induce systemic inflammation and sepsis when it is present in an excessive amount (58). The permeability of BBB in germ-free mice decreased after LPS administration (56). In addition, a study reported that systemic LPS can stimulate the microglia, resulting in chronic neuroinflammation in germ-free mice (59). Moreover, indigestible diet fibers and resistant starch can be fermented by gut microbiota, producing SCFAs (such as acetate, propionate, and butyrate) and other metabolites such as hydrogen and methane (60). SCFAs affect the psychological functions and suppress inflammation by affecting cellular functions including G-protein-coupled receptor activation and histone deacetylase activation, which further affect host intestinal epithelial integrity, BBB integrity, and brain functions (61–63). The gut-derived TMA, namely, trimethylamine-n-oxide (TMAO), is secreted by microbiomes such as *Anaerococcus*, *Clostridium*, *Desulfovibrio*, and *Providencia* (64). The presence of TMAO in cerebrospinal fluid revealed its ability to cross the BBB (65). Interestingly, in a clinical study, the TMAO levels in the cerebrospinal fluid increased in cognitively impaired individuals with AD, representing a finding that may be useful for developing a therapeutic approach for the NDs characterized by protein misfolding such as AD (66).

#### 1.3.3 Nervous System Modifications

The bidirectional communication network comprises the CNS, autonomic nervous system (ANS), enteric nervous system (ENS), and the hypothalamic–pituitary–adrenal (HPA) axis.

Microbiota communicates with the brain *via* the vagus nerve. The absence of specific neurochemical and behavioral effects in vagotomized germ-free mice demonstrated that the vagal pathway is an important communication route between the gut and the brain (37). The ENS interacts with the CNS through the vagus nerve by generating direct neurochemical signals from the gut microbiota to the brain and *vice versa* (67). The HPA axis is a part of the limbic system having structures such as the hippocampus, hypothalamus, and amygdala and involving memory and emotional responses. Chronic stress or pro-inflammatory cytokines such as interleukin (IL)-6 increase the level of corticotropin-releasing factor secreted from the hypothalamus and adrenocorticotropic hormone (ACTH) secreted from the pituitary gland, which results in the secretion of cortisol from the adrenal gland, which is toxic to the brain (68).

Consequently, the combination of neural and hormonal communications facilitates the CNS to influence the activities and function of intestinal cells (69, 70). Moreover, gut microbiota affects host health by modulating gut cells and maintaining intestinal metabolic and immune homeostasis (71–73). For example, microbial intestinal dysbiosis and increased intestinal permeability associated with *Clostridium* overgrowth are considered a feature of immune-related intestinal and extra-intestinal disorders (74).

Interestingly, the microbiota also alters the production of neurotransmitters and hormones such as dopamine, adrenaline, noradrenaline, serotonin (5-HT), gamma-aminobutyric acid (GABA), glucagon-like peptide-1, and peptide YY or their precursors, which act on the CNS or ENS directly *via* the vagus nerve or indirectly by entering the circulation (**Figure 1**) (38, 75).

#### 1.3.4 Modification of Immune System

Gut microbiota plays an indispensable role in the maturation of the host immune system and intestinal homeostasis (4). The dysfunction of the interaction between microbiota and immune system induces immune signaling, thus indicating implications in CNS development and NDs (24). Studies have shown that the gut microbiota is associated with the regulation of some immune signaling pathways such as the inflammasome signaling pathway, type I interferon signaling pathway (IFN-I), and nuclear factor (NF)-κB signaling pathway (18, 76).

Compared with the wild-type mouse model, the ASC-, caspase-1-, and IL-18 (typical inflammasomes) knockout mouse model showed altered α-diversity in a study (77). Furthermore, evidence suggests that the activated inflammasome and the increased level of pro-inflammatory cytokines such as IL-1β, IL-6, and IL-18 proteins are associated with the major depressive disease (78). In MS, inflammasome signaling can be inhibited by IFN (79). IFN-I is associated with the maturation of dendritic cells (DCs), enhancement of cytotoxic T cells, and bidirectional interaction between the host and the gut microbiota (80). However, commensal lactic acid bacteria can trigger the Toll-like receptor (TLR) 3-mediated IFN-I secretion of intestinal DCs (81). In addition, because the critical transcription factor contributes to immune response, the increased NF-κB level with the cooperative expression of TNF-α was detected both in the intestinal and hippocampal zones, which are associated with amnesia; the symptoms of amnesia and colitis were attenuated after the recovery of gut microbiota α-diversity was disturbed in a colitis model (82).

## 2 From Bench to Clinic: The Emerging Role of Gut Microbiota in Neurodegenerative Diseases

**Figure 2:**
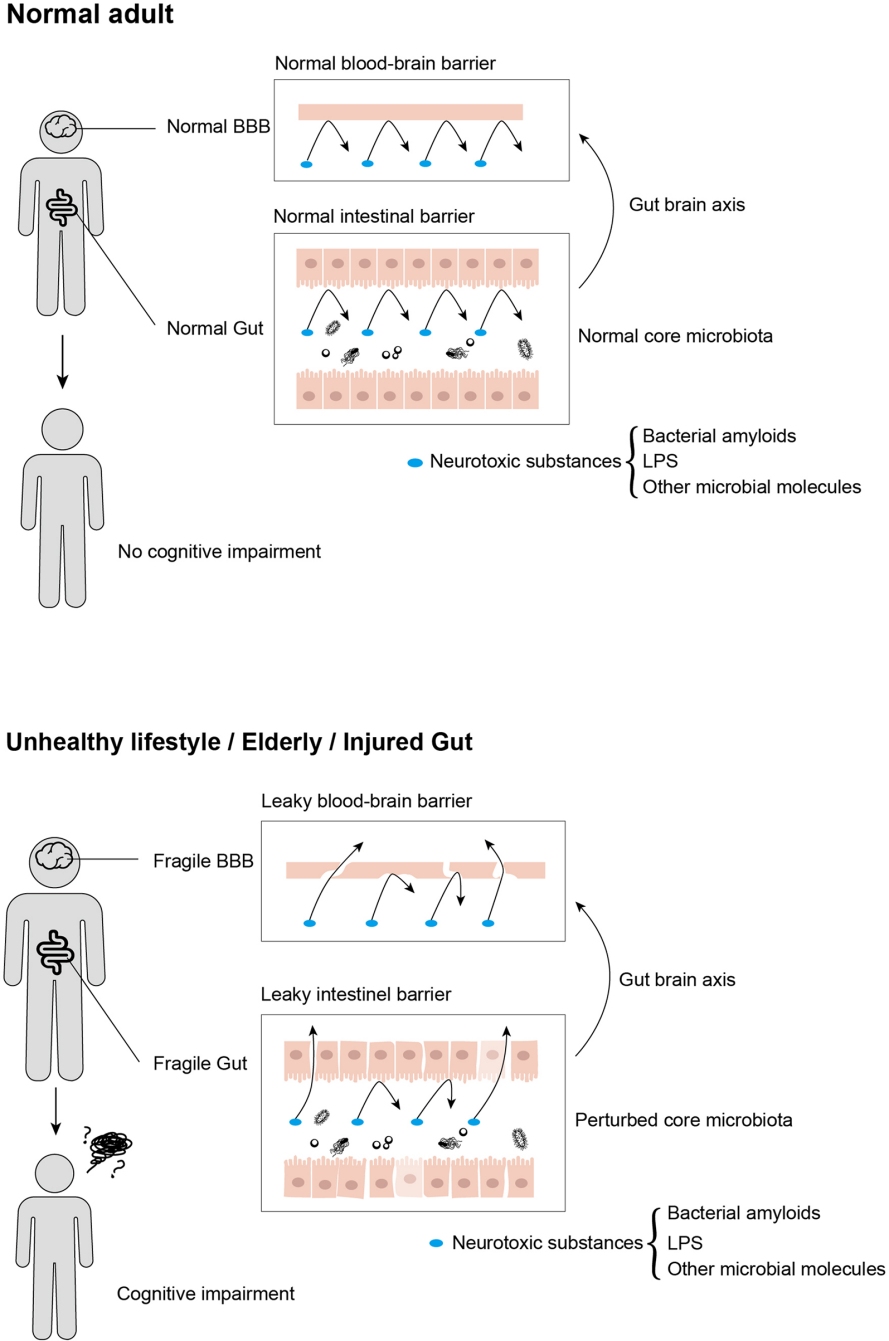
Neurotoxic substances that are produced by gut microbiota influence cognitive impairment progression by transmitting from gut to brain, in which intestinal barrier and blood–brain barrier served as crucial customs passes. Moreover, these neurotoxic substances cannot traverse from usual intestinal and blood–brain barriers, while aging, unhealthy lifestyles, and acute or chronic enteritis would disrupt the integrity of both intestinal and blood–brain barriers, leading to cognitive impairment.

Gut dysbiosis has adverse effects on cognition, behavior, and motor performance (83). The frailty of the gut on the physiology alters the intestinal environment and gut microbiota (**Figure 2**) (22). The gut microbiota, being considered as potential diagnostic features of NDs, affects different pathophysiological stages in cognitive impairments. In this section, we review the involvement of microbiota in typical NDs (**Table 1**).

**Table 1:** Involvements of gut microbiota in neurodegenerative disease.

Bacterial genus	Possible involvements	Related neurodegenerative diseases	Reference
*Escherichia, Pseudomonas, Staphylococcus, Streptococcus, Bacillus, Mycobacteria, Citrobacter, Klebsiella, Salmonella*	Bacterial amyloid, FapCs, Translocate across BBB through GBA	Alzheimer’s disease	Cao et al. (84)
*Lactobacillus*	GABA, Balance the regulation of cortical excitability and neural excitation-inhibition	Alzheimer’s disease	Ciminelli et al. (85) Auger et al. (86)
*Bifidobacterium*	GABA, Balance the regulation of cortical excitability and neural excitation-inhibition	Alzheimer’s disease	Auger et al. (86)
*Proteobacteria,* *Bacteroidetes,* *Firmicutes,* *Actinobacteria,* *Lachnospiracea*	Unknown	Alzheimer’s disease	Vogt et al. (87) Zhuang et al. (88) Ling et al. (89)
*Roseburia,* *Faecalibacterium*	Can produce SCFAs	Parkinson’s disease	Nuzum et al. (90)
*Pseudomonas*	Fap, change of α-synuclein	Parkinson’s disease	Christensen et al. (91)
*Enterobacteriaceae*	Curli, α-synuclein aggregation	Parkinson’s disease	Sampson et al. (92)
*Clostridium coccoides, Bacteroides fragilis,* *Prevotellaceae*	Unknown	Parkinson’s disease	Hopfner et al. (93) Hasegawa et al. (94) Scheperjans et al. (95)
*Hydrogen-product bacteria*	Reduced dopaminergic loss	Parkinson’s disease	Fujita et al. (96) Yorikata et al. (97) Guo et al. (98)
*Coriobacteriales, Erysipelotrichales, Bacteroidales, Burkholderiale*	Unknown	Huntingdon disease	Kong et al. (99) Radulescu et al. (100)
*Clostridium*	Decrease level of SCFA secretion	Multiple sclerosis	Miyake et al. (101)
*Firmicutes, Bacteroidetes,* *Prevotella*	Unknown	Multiple sclerosis	Cosorich et al. (102) Chen et al. (103)

BBB, blood–brain barrier; GBA, gut–brain axis; GABA, gamma-aminobutyric acid; SCFA, short-chain fatty acid.

### 2.1 Alzheimer’s Disease

AD is the most common ND in the elderly population, and age-related adult dementia accounts for 60%–70% of dementia cases; the lifetime AD risk is approximately 20% in women and 10% in men (104). AD is chronic and irreversible and involves progressive cognitive impairment and behavioral changes such as memory loss, disorientation, and loss of mobility, which are characterized by synaptic dysfunction by synthesized factors, accumulation of neurotoxic protein aggregates, age-related processes, neuroinflammation, lead neuron, and synaptic loss (105–107). A classical pathology of AD involves amyloid-beta (Aβ) extracellular neurotic plaques, which are distributed throughout the cerebral cortex, and over-phosphorylated Tau protein-containing neurofibrillary tangles, which primitively occur in the medial temporal lobe and then diffuse to the isocortical regions of the temporal, parietal, and frontal lobes (108–110). Studies have reported that the deposition of Aβ and Tau protein occurs 10–20 years before the onset of clinical dementia symptoms (111). Numerous studies on AD treatment or intervention strategies in animal models have achieved promising results. Unfortunately, drugs targeting the pathological procedure have been found not effective in AD clinical treatment (112).

#### 2.1.1 Involvement of Microbiome in Alzheimer’s Disease

The frailty of the host is related to the reduced diversity of core microbiota groups such as *Lactobacilli*, *Bacteroides*, and *Prevotella* and the increased abundance of *Ruminococcus*, *Atopobium*, and *Enterobacteriaceae* (113). Interestingly, these microbial communities are associated with the host’s mood and behavior, which are precipitating factors for cognitive impairment. Studies have reported that gut microbiota is altered in AD. When AD patients were compared with healthy controls, AD patients exhibited diverse microbiota, an increased abundance of *Bacteroidetes*, and a reduced abundance of *Firmicutes*, *Proteobacteria*, and *Actinobacteria* (88, 89). Moreover, the composition of the gut microbiota of SAPM8 mice, which exhibited learning and cognitive impairment similar to AD patients, revealed conspicuous character divergence compared with that of the healthy control; the correlation density and clustering operational taxonomic unit of gut microbiota decreased (87). The increasing abundance of microbiota such as *norank f Lachnospiracea*, *unclassified f Lachnospiraceae*, and *Alistypes* in SAPM8 models is consistent with that reported in AD patients in another study (114). The analogous shifts of the microbiota were also found in other transgenic AD pathology-like mouse models compared with 3-year-old wild-type mouse models; the microbiota composition was similar but the diversity started changing after 6 months of age, and the AD pathology-like models showed increased abundance of *Proteobacteria* and *Erisilopelotrichaeae* (115). In addition, neuroinflammation and amyloidosis are affected by the perturbation of gut microbiota diversity induced by antibiotics in the AD mouse model (116, 117).

**Figure 3:**
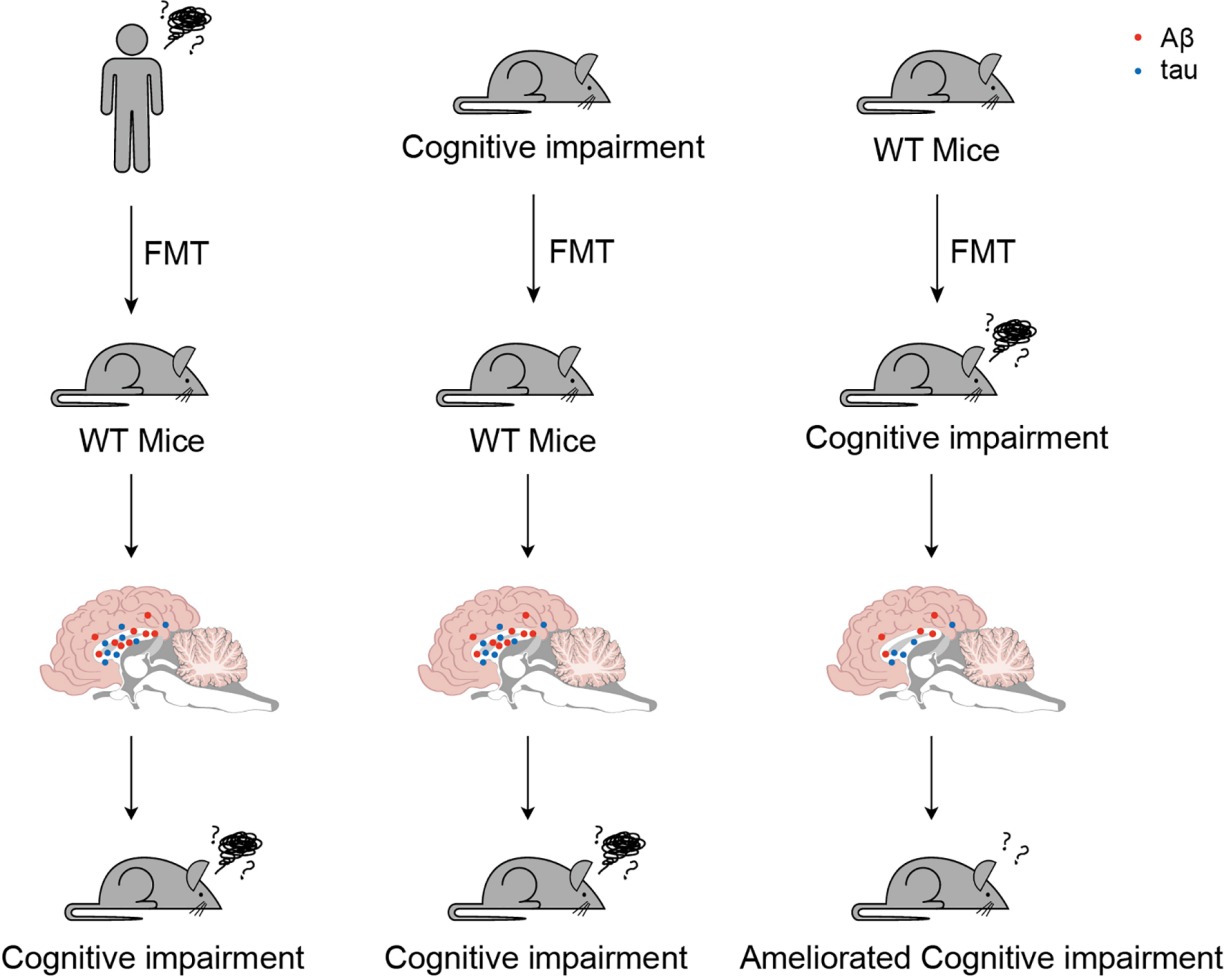
Served as both a typically investigative and a potential therapeutic approach for cognitive impairment, utilize fecal microbiota transplantation (FMT) has been spread. These three typical trials suggested potential prevention or clinical therapies for cognitive impairment.

Fecal microbiota transplantation (FMT) is considered both a typically investigative and a potential therapeutic approach for ND (**Figure 3**). A study reported increased cerebral Aβ pathology in a germ-free amyloid precursor protein (APP) transgenic mouse model with FMT compared with that in a cognitively impaired mouse model, whereas wild-type mice with FMT exhibited less effective pathology (118). Compared with germ-free mice with FMT from AD patients and healthy donors, the FMT model showed cognitive impairment (119). Correspondingly, frequent FMT from healthy wild-type mouse models to AD pathology-like transgenic mice effectively reactivated the glial cells and reduced Aβ pathology, neurofibrillary tangles, and cognitive impairment (120).

#### 2.1.2 Modifications of Microbial Molecules in Alzheimer’s Disease

As previously mentioned, the gut microbiota-derived molecules may cause gut dysbiosis, and GBA is a crucial precipitating factor for AD. LPS, peptidoglycan (PGN), bacterial epigenetics, bacterial DNA, and bacterial amyloids are the typical MAMPs associated with AD. The cluster differentiation 14 (CD14) receptor, termed LPS receptor, coordinates with the microglia to promote A_β_ deposition, stimulates TLR4 in astrocytes that is a ligand for A_β_ metabolism, and accelerates neuron loss (58, 121, 122). AD patients showed 2-fold higher levels of LPS in the neocortex and 3-fold higher LPS levels in the hippocampus than healthy controls (123). Andreadou et al. (124) reported an increased level of LPS in both cerebrospinal fluid and serum in AD patients and a negative correlation between the LPS level and cognitive state. In AD mouse models, LPS induced cognitive impairment, neuroinflammation, and sickness behaviors such as anxiety and fear (125, 126). PGN is a dominant component of the Gram-negative cell wall that is recognized by specific pattern-recognition receptors (PRRs) of the innate immune system (127). Gut microbiota-derived PGN could traverse the BBB and affect gene transcription and social behaviors (128). Currently, the pathogenic role of bacterial DNA in AD is being considered. In *in vitro* AD pathological assays, Tetz et al. (129, 130) reported the induction of Tau aggregation, as well as Aβ misfolding and aggregation by bacterial eDNA. Bacterial amyloids are the extracellular proteins secreted by *Escherichia*, *Pseudomonas*, *Staphylococcus*, *Streptococcus*, *Bacillus*, *Mycobacteria*, *Citrobacter*, *Klebsiella*, and *Salmonella* species and could translocate across the BBB through GBA associated with NDs (84). In *in vitro* assays, specific bacterial amyloid FapCs were found to be Aβ-binding hot spots that participate in the incorporation of Aβ nanofibrils (74). In zebrafish AD models, fragment amyloids from infectious bacteria enhanced Aβ pathogenesis and further made the cognitive impairment more severe (131). The crucial role of SCFA in AD has emerged. In an *in vitro* trial, SCFA inhibited A_β_ aggregation (132). By comparing the AD mouse model and wild-type mouse model of different ages, the perturbed α-diversity of microbiota and decreased level of SCFAs were found to be associated with the bacterial amyloid deposition and ultrastructural alteration in the gut (133). In a similar trial by Zheng et al. (134) on the content of SCFAs in fecal samples, the AD mouse model showed a significant difference in the levels of propionic acid, isobutyric acid, 3-hydroxy butyric acid, and 3-hydroxisopropyl acid as well as the decreased levels of lactic acid, 2-hydroxy butyric acid, 2-hydroxy isobutyric acid, levulinic acid, and valproic acid compared with wild-type mice. In addition, an *in vitro* study suggested that sodium butyrate could protect the neuron cells from Aβ-induced neurotoxic effects (135).

In co-metabolism by host and gut microbiota, bile acids maintain the secondary function of steroid hormones by serving as signaling molecules that affect the cellular receptors associated with CNS development (136), including membrane-bound receptors (such as sphingosine-1-phosphate receptor 2 and Takeda G-protein-coupled bile acid receptor 5) and nuclear receptors (such as Farnesoid X receptor) (137) In a study of 1,562 clinical cases, diverse bile acid metabolites in serum were quantified, which revealed that bile acids are the biomarker of AD pathology (138).

TMAOs are the metabolites of dietary choline. In the network-based algorithm engineered by Xu and Wang (139), TMAO ranked first in 56 human AD biomarkers. The increase in TMAO is detectable in patients with cognitive impairment (65). In an *in vitro* trial, researchers observed that TMAO participates in Aβ aggregation (140). In AD mouse models, TMAO administration accelerated the senescence of hippocampal cells and Aβ pathology and aggravated cognitive impairment (141). In another study, long-term cognitive impairment was ameliorated with reduction in the TMAO proportion in the plasma of the AD mouse model (142). This finding suggested that TMAO exacerbates ND pathology and cognitive impairment; however, the changes in serum TMAO in ND patients remain to be investigated.

GABA, the precursors of which are glutamates metabolized by the genera *Lactobacillus* and *Bifidobacterium*, is an essential inhibitory neurotransmitter, which balances the regulation of cortical excitability and neural excitation-inhibition. GABA plays a crucial role in CNS development (85). A study reported that the inhibitory GABA signaling could ameliorate cognitive impairment in the cognitive impairment mouse model (86). The disruption of GABA balance is considered a contributor to cognitive impairment, warranting further studies.

### 2.2 Parkinson’s Disease

PD is a progressive ND, with aging as the main risk factor, and its estimated morbidity is 6% (143, 144). PD can be manifested by both motor and non-motor symptoms. Motor symptoms include resting tremor, bradykinesia, postural instability, and rigidity. For some PD patients, parkinsonian tremor is the only visible symptom during diagnosis (145). Non-motor symptoms include cognitive decline, depression, anxiety, dysautonomia, dementia, and sleep disturbances (145). PD is closely related to gastrointestinal complications such as bloating, nausea, and abdominal discomfort (146).

One of the general pathologies of PD is the loss of dopaminergic neurons in the substantia nigra pars compacta (SNpc) located in the midbrain, which is mainly responsible for motor disorders. The degeneration of dopaminergic neurons is closely associated with Lewy bodies, which are cytoplasmic inclusions that comprise insoluble alpha-synuclein (α-Syn) aggregates (147). The six stages of such Lewy pathology in PD have been demonstrated. The disease was proposed to start in the gut with misfolded α-Syn and then be localized to the brain (148).

#### 2.2.1 Involvement of Microbiome in Parkinson’s Disease

Despite intensive studies on PD, no effective treatments with sustained benefits are available. Recent findings have shown that gut microbiota is closely related to PD and causes changes in microbe diversity and metabolites. In several case–control studies, an increasing abundance of *Lactobacillaceae*, *Barnesiellaceae*, and *Enterococcacea* was observed and a decreasing abundance of *Clostridium coccoides*, *Bacteroides fragilis*, and *Prevotellaceae* have been observed in PD patients compared with those in healthy controls (93–95). PD patients may also have increased intestinal permeability and bacterial overgrowth in the small intestine (149, 150). A recent study in the α-Syn-overexpressing (ASO) mice showed that the gut microbiota plays a crucial role in PD manifestation. ASO mice administered feces from PD patients showed increasing motor symptoms compared with the mice administered healthy feces (151). A case report of a PD patient with healthy FMT showed the temporary improvement of leg tremors and other PD symptoms (152).

#### 2.2.2 Modifications of Microbial Molecules in Parkinson’s Disease

Gut-derived microbial molecules are considered crucial biomarkers of PD, in addition to AD. LPS acts as a PD-inducing factor that causes intraneural LPS to activate microglia and dopaminergic neuron degeneration. In a mouse model, microglial nicotinamide adenine dinucleotide phosphate oxidase expression was shown to be regulated by LPS, leading to mitochondrial dysfunction, which further initiated neurotoxic effects (153, 154). Currently, LPS administration is extensively used to induce PD-like pathology in mouse models (155). PGNs are recognized as exogenous foreign substances by the host immune system, and they are known as ligands with PRRs because they are unique to bacteria (127). A recent study on the PGN recognition protein genes suggested its causative role in gut microbiota and gut homeostasis related to PD risk (156, 157).

Moreover, two types of bacterial amyloids, namely, Fap and Curli, are associated with PD pathology. Fap produced by *Pseudomonas* induced a conformational change in α-Syn in an *in vitro* trial (91). Curli produced by *Enterobacteriaceae* promoted α-Syn aggregation and motor impairment in a mouse model (92).

After comparing fecal samples of patients with those of healthy controls, patients with PD showed SCFA reduction and altered microbiota composition (57). Interestingly, the plasma SCFA levels increased with the severity of PD and antiparkinsonian medical approaches (158). In addition, the colonization of SCFA-producing bacteria such as *Roseburia* and *Faecalibacterium* was found to be more in healthy controls than that in patients with PD (90).

Whether TMAO can be used as a diagnostic biomarker of PD is unknown. However, it was detectable in the CSF of a PD mouse model (141). TMAO level alterations in PD are still controversial because its high plasma levels were associated with terminal PD, whereas low plasma TMAO levels were associated with increased risk of early-stage PD (159, 160).

Molecular hydrogen is a common by-product of carbohydrate fermentation in the host microbiota. Due to the bidirectional translocation from the cell membrane and antioxidant properties, molecular hydrogen might have neuroprotective effects and is being used in bacterial overgrowth in the small intestine (161). A clinical study revealed the low abundance of bacterial hydrogen products in patients with PD compared with that in healthy controls (162). Inflammation and peripheral blood cell apoptosis in healthy adults can be reduced by drinking hydrogen-rich water (163). Moreover, in a previous study using a PD mouse model, dopaminergic loss was shown to reduce with drinking of hydrogen water (96). Moreover, regular drinking of hydrogen water reduced motor impairment in patients with PD (97). A similar study using an autism spectrum disorder mouse model also reported amelioration of the autistic-like behavior in mice (98). However, the specific mechanism of hydrogen in modulating cognitive impairment is still unknown.

### 2.3 Huntington Disease

HD is an autosomal dominant rare ND with an estimated global prevalence of 2.7 cases in 100,000 people, with the onset age between 35 and 44 years (164, 165). The main manifestations of HD include cognitive impairment, psychiatric disorder, and motor symptoms. Motor disturbance progresses into dysphagia with weight loss and aspiration difficulties, leading to fatality (164, 166). In addition, the abnormal expansion of *HTT* results in HTT dysfunction in brain development, transcriptional process, histone modification, and mitochondrial function, which eventually triggers the aforementioned manifestations (165, 167). Despite the availability of explicit information on HD symptoms and pathogenesis, no effective treatment is available for curing the disease or delaying its progression.

#### 2.3.1 Involvement of Microbiome in Huntington Disease

Emerging evidence has linked gut microbiota with neurological health, thus creating the possibility of bringing gut microbiota into HD diagnosis and treatment (168). HD may be characterized by changes in the abundance or diversity of gut microbiota, and such changes may include sexual differences (169). A recent study compared the gut microbiota in an HD mouse model with that in wild-type mice and reported an increase in the abundance of *Bacteroidales* and *Lactobacillales* and a decrease in the abundance of *Clostridiales* in a male HD mouse model. By contrast, an increase in the abundance of *Coriobacteriales*, *Erysipelotrichales*, *Bacteroidales*, and *Burkholderiale* and a decrease in the abundance of *Clostridiales* were observed in female HD mice. Furthermore, male HD mice showed higher microbial diversity than both female and wild-type mice (99). Another study showed the decreased levels of myelin-related proteins and mature oligodendrocytes in the prefrontal cortex in microbiota-deficient mice, which led to reduced callosal myelination and white matter plasticity (100); the study revealed the effect of the lack of microbiota in aggravating internal HD phenotypes.

#### 2.3.2 Modifications of Microbial Molecules in Huntington Disease

In addition to the relation between HD and gut microbiota diversity, some SCFAs and bioactive metabolites derived from gut microbiota secretion are evident in HD onset and progression, which mainly act on the biological processes of the GBA (170). Serotonin, tyrosine, 2-hydroxyphenylacetic acid, 3-hydroxyphenylacetic acid, and 4-hydroxyphenylacetic acid can cause diet and bioactive compound dysbiosis in the GBA, whereas indole-3-propionic acid can lead to intestinal permeability (171). More studies on such gut microbiota-derived metabolites can help in understanding the complex relationship between gut microbiota and HD and shed light on the early diagnosis and treatment of HD.

### 2.4 Multiple Sclerosis

MS is a chronic inflammatory and demyelinating disorder in the CNS. MS exhibits different phenotypes, with approximately 15% cases in a primary progressive MS (PPMS) course and 85% cases in a relapsing-remitting MS (RRMS) course (172). PPMS is a progressive neurologic disorder characterized by spastic paraparesis and sphincter dysfunction (173). A patient presenting with clinically isolated syndromes can be suspected as having RRMS, which is characterized by sustained neurologic symptom relapse and recovery (174, 175). The essential pathology of MS remains to be elucidated. However, MS development may be affected by both internal and external factors, eventually leading to immune dysregulation.

#### 2.4.1 Microbiota Implicates Internal Factor Effect on Multiple Sclerosis

Recent studies have shown that internal gut microbiota significantly effects MS and can be affected by environmental factors (176). The 16S rRNA sequencing of intestinal microbiota showed a high abundance of the phylum Firmicutes and lower abundance of the phylum Bacteroidetes in patients with MS than those in healthy controls (102). The increased abundance of *Euryachaeota* and *Akkermansia* has been observed in untreated patients with MS compared with that in healthy controls (177). Other studies have pointed out specifically that the decreased abundance of *Prevotella* in patients with RRMS can increase the disease activity (103). The reduction in *Clostridium* abundance in patients with RRMS, leading to a decreased level of SCFA secretion, was also observed in a study (101). Considering the extent of such changes in patients with MS, FMT trials have further proven the relationship between gut microbiota and MS. The transplant of MS microbiota in mouse models resulted in an increased experimental autoimmune encephalomyelitis incidence, resulting in more severe MS symptoms (178, 179). Currently, two human FMT trials have reported the successful amelioration of MS symptoms (180, 181).

#### 2.4.2 Microbiota Implicates External Factor Effects on Multiple Sclerosis

External environmental factors such as Epstein–Barr virus infection, smoking, and vitamin D intake also have significant effects on MS progression. The relationship between vitamin D and MS has led to research on an upsurge in the effect of diet in MS treatment (182). Diets can affect the diversity and levels of gut microbiota and then indirectly affect MS development (183). Patients with obesity showed the same phenomenon of increase in Firmicutes and Actinobacteria abundance similar to those in patients with MS. Additionally, obese patients with MS had a reduced abundance of Bacteroidetes compared with patients having normal weight (184). Moreover, obese patients exhibit low levels of 25-hydroxyvitamin D3 (vitamin D storage form) and consequently high risks of MS development (185). Dietary studies in patients with MS have shown the positive effects of dietary intervention with vitamin D supplement in low-calorie diets, alleviating the chronic inflammatory symptoms of MS (186). Recently, intermittent fasting has been introduced into MS treatment because of its significant effects, including the supply the abundant of gut microbiota and the secretion of leptin and glutathione (187). All these studies have shown that diet can be considered as an MS treatment alternative.

## 3 Controversies and Perspectives

NDs are systemic diseases that can be studied in diverse disciplines including microbiology and neuroscience. Several pieces of evidence in preclinical, clinical, *in vitro*, and *in vivo* studies have suggested the relationship of gut microbiota with brain shapes, neurological processes, and cognitive behavior (**Figure 2**). However, the findings of most studies are less reliable because of limited sample sizes. The effect of microbiota on ND pathophysiology in FMT trials is unclear because of the unreliable microbiota, and most trials have focused on animal models.

Comprehensive, normalized, and rigorous analysis and evaluation standards are needed because of differences in cohorts, lifestyles, ages, and genders across studies. The differences across studies can be summarized as follows.

First, tremendous regional differences have been observed, for example, the abundance of gut Bacteroides was reported to be high in AD patients from the USA, whereas it was reported to be low in AD patients from China (87, 188). Two studies from different provinces in China also showed an inverse change in *Blautia* abundance (188, 189). Second, a few studies have reviewed the effects of specific species of microbiota on ND pathology; however, the mechanisms remain unclear. For example, the abundance of *Akkermansia* increased in human patients with AD, PD, and MS, whereas *Akkermansia* intragastric administration in an AD mouse model produced protective effects on cognitive impairments; increased *Akkermansia* proportion in AD and PD mouse models has also been reported to ameliorate the underlying pathology (87, 177, 190–192). Based on the aforementioned findings, we speculate that controversial experimental results were obtained because of the limited pathogenesis of ND mouse models. Human ND etiologies are quite intricate, involving long-term manifold metabolic disorders, gut microbiota alteration, gene mutation, and various hereditary factors, whereas most mouse model etiologies rely on gene editing or medical injection, which might lead to inconsistent pathogenic processes (24, 193–201). Third, studies have demonstrated that microbiota altered with aging; yet, no evidence is available to confirm whether the altered microflora is healthy, unhealthy, stable, or vulnerable (10, 15, 22, 113, 202). Construction of a standard ND patient fecal microbiota bank can revolutionize the analysis of fecal microbiota through a noninvasive ND diagnostic approach. Furthermore, more precise animal models that simulate both human ND pathology and its intestinal environments are needed.

Similarly, the nervous system and BBB become weaker with aging (49, 203, 204). Thus, it is essential to understand how the vulnerability of neurons and BBB affects GBA modulation. Further studies are required to assess the long-term effect of GBA stability as a new “endocrine organ” on ND pathology (48). Specific microbial molecule modulation in gut–brain signaling both chemically and physically may provide a therapeutic approach targeted on microbiome effects. For example, the application of microbiota-targeted psychotropic medicines without dependency, which could reduce the dependency caused by existing psychotropic drugs or the drugs that inhibit the proliferation of specific ND-causing microbiota or bacterial metabolite production, might be a revolutionary therapy.

## 4 Conclusion

Gut microbiota influences brain disorders through modulating the immune system, direct neural signaling, and activating the humoral pathway by microbial molecules and some unknown potential pathways. Considerable attention has been paid toward elucidating the unknown mechanism and influence factors; therefore, direct intervention of ND pathophysiology by gut microbiota should be reconsidered. Further studies from bench to clinical on these mysteries are required to better understand the underlying mechanism.

## Author Contributions

HZ and YC wrote the first draft of the article and contributed equally to this work. WW, ZW, GX, ML, and BY contributed substantially to the writing and revised the article. HC designed the figures. WW and PC designed the editorial aim. All authors have made substantial intellectual contributions, are responsible for the paper, and ultimately endorse the submitted version. WW has the primary responsibility for final content.

## Funding

This work was supported by the National Natural Science Foundation of China (31872442) and Key Realm R&D Program of Guangdong Province (2020B0202080002).

## Conflict of Interest

The authors declare that the research was conducted in the absence of any commercial or financial relationships that could be construed as a potential conflict of interest.

## Publisher’s Note

All claims expressed in this article are solely those of the authors and do not necessarily represent those of their affiliated organizations, or those of the publisher, the editors and the reviewers. Any product that may be evaluated in this article, or claim that may be made by its manufacturer, is not guaranteed or endorsed by the publisher.
